# A systematic review and meta-analysis of angiotensin-converting enzyme inhibitor use and psoriasis incidence

**DOI:** 10.1038/s41598-021-89490-z

**Published:** 2021-05-11

**Authors:** Gonjin Song, Ji Yea Kim, Ha Young Yoon, Jeong Yee, Hye Sun Gwak

**Affiliations:** College of Pharmacy and Graduate School of Pharmaceutical Sciences, Ewha Womans University, 52 Ewhayeodae-gil, Seodaemun-gu, Seoul, 03760 Republic of Korea

**Keywords:** Adverse effects, Drug therapy

## Abstract

Although a considerable volume of data supporting induction or aggravation of psoriasis because of angiotensin-converting enzyme (ACE) inhibitor use exists, it remains insufficient for definitive conclusions. Therefore, we aimed to evaluate the association between ACE inhibitor use and psoriasis incidence through a systematic literature review and meta-analysis. We searched for qualifying studies across PubMed, Web of Science, and Embase. Odds ratios (ORs) and 95% confidence intervals (CIs) were calculated to evaluate the strength of the association between ACE inhibitor use and psoriasis incidence. Eight studies with a total of 54,509 patients with a psoriasis diagnosis were included in this meta-analysis. The pooled OR for psoriasis incidence among ACE inhibitor users was 1.52 (95% CI, 1.16–2.00) compared to that among non-users. From subgroup analysis by continent, the OR for ACE inhibitor users versus non-users was 2.37 (95% CI 1.28–4.37) in Asia. Per the subgroup analysis by climate, the OR for ACE inhibitor users vs non-users in dry climate was 3.45 (95% CI: 2.05–5.79) vs 1.32 (95% CI 1.01–1.73) in temperate climate. Our results reveal a significant association between ACE inhibitor use and psoriasis incidence.

## Introduction

Angiotensin-converting enzyme (ACE) inhibitors are widely used medications for treatment of hypertension and congestive heart failure and prevention of kidney diseases in patients with hypertension or diabetes^1^. Although ACE inhibitors are generally well-tolerated, several adverse events have been reported, including dry cough, angioedema, rash, and hyperkalemia^2^. In addition, psoriasis has been reported as a rare adverse event. Several case reports have implicated the use ACE inhibitors, particularly captopril and enalapril, in the incidence and exacerbation of psoriasis^1,3–9^. Moreover, psoriasiform eruptions and exacerbation of pre-existing psoriasis were reported in patients receiving other ACE inhibitors such as ramipril and lisinopril^3,5^.

Therapeutic agents which may induce or aggravate psoriasis may be classified into three categories depending on the strength of the association. First group of drugs have a strong association with psoriasis incidence or aggravation and include lithium, β-blockers, and antimalarials. Second group of drugs have considerable yet insufficient data supporting incidence or aggravation of psoriasis, such as ACE inhibitors, interferons, and terbinafine. Third group of drugs have been occasionally reported to be associated with incidence or aggravation of psoriasis and include a large group of miscellaneous drugs such as clonidine, digoxin, and amiodarone^10^.

Although studies reporting the possible association between ACE inhibitor use and psoriasis exist, the results have been inconsistent. Therefore, we investigated the association between ACE inhibitor use and psoriasis incidence through a systematic literature review and meta-analysis. In addition, because environmental factors may affect the association between ACE inhibitor use and psoriasis, subgroup analyses by study design, continent and climate were also performed.

## Results

### Identification and characteristics of the included studies

**Figure 1:**
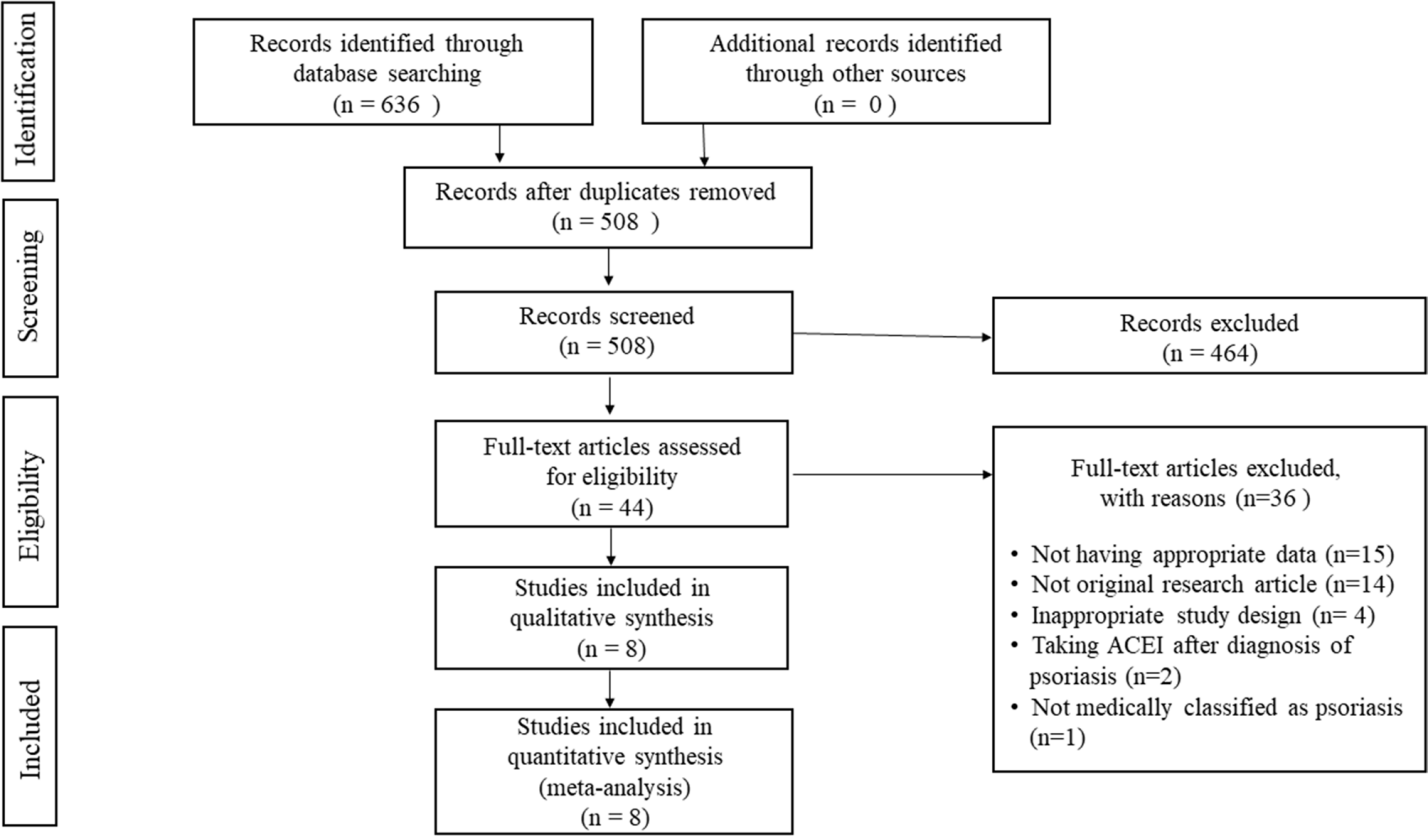
Flow diagram of the study selection process.

The study selection process is summarized in Fig. 1. In total, 636 records were identified from the three databases (PubMed n = 201, Web of Science n = 60, Embase n = 375), and 128 duplicates were excluded. After removing 464 studies during title and abstract screening, 44 studies were selected for full-text review, and 36 studies were excluded for the following reasons: not having appropriate data (n = 15), not original articles (n = 14), not case–control or cohort studies (n = 4), not confirmed whether drugs were taken before diagnosis (n = 2), and not medically classified as psoriasis (n = 1). Finally, eight studies were included for meta-analysis^11–18^.

The characteristics of the included studies are shown in Table 1. Of the eight studies, four were cohort studies^15–18^ and four were case–control studies^11–14^. Four studies were performed in Asia^11,14,17,18^, three in Europe^12,13,16^, and one in the USA^15^. The number of patients in each study ranged from 625 to 256,356. Regarding study quality using the Newcastle–Ottawa Scale (NOS) score, each study received ≥ 6 points out of 9 (Table 1).

**Table 1:** Characteristics of studies included in the systematic review.

References	Country	Study design	Definition of patients with psoriasis	No. of patients with psoriasis (male %)	No. of population (male%)	Age (years) mean ± SD)	Major comorbidities (% of all patients with psoriasis)	NOS
Cohen et al. ^11^	Israel	Case–control study	Patients who were hospitalized for extensive psoriasis	110 (57.3)	625 (58.9)	Case: 49.6 ± 17.2 Control: 53.4 ± 17.0	NA	6
Gerdes et al. ^12^	Germany	Case–control study	Patients with severe psoriasis	1131 (NA)	8230 (NA)	49.7 ± 16.6	Hypertension (25.3%) Diabetes mellitus (9.2%) Hyperlipoproteinaemia (7.7%)	7
Brauchli et al. ^13^	UK	Case–control study	Patients with an incident psoriasis diagnosis	36,702 (46.2)	73,404 (46.2)	NA	Hypertension (14.4%) Ischemic heart disease (7.1%) Hyperlipidaemia (5.9%)	8
Al-Mutairi et al. ^14^	Kuwait	Case–control study	Patients with psoriasis vulgaris	1835 (52.5)	3670 (52.5)	case: 52.3 ± 11.9 control: 52.7 ± 13.5	Diabetes mellitus (39.2%) Hypertension (32.6%) Metabolic syndrome (16.7%)	7
Wu et al. ^15^	USA	Prospective cohort study	Patients with physician-diagnosed incident psoriasis	817 (0)	77,728 (0)	NA	Cardiovascular disease Type 2 diabetes Hypercholesterolemia Hypertension	6
Jacob et al. ^16^	Germany	Retrospective cohort study	Patients with an incident psoriasis diagnosis	NA	144,296 (48.6)	68.7 ± 12.7	Hyperlipidemia	7
Kim et al. ^17^	Korea	Retrospective cohort study	Patients with an incident psoriasis diagnosis	9254 (NA)	256,356 (51.4)	NA	Hypertension Diabetes mellitus Dyslipidemia	8
Liu et al. ^18^	Taiwan	Retrospective cohort study	Patients with an incident psoriasis diagnosis	89 (100)	10,282 (100)	NA	Prostate cancer	6

*NA* not applicable.

### Quantitative data synthesis

**Figure 2:**
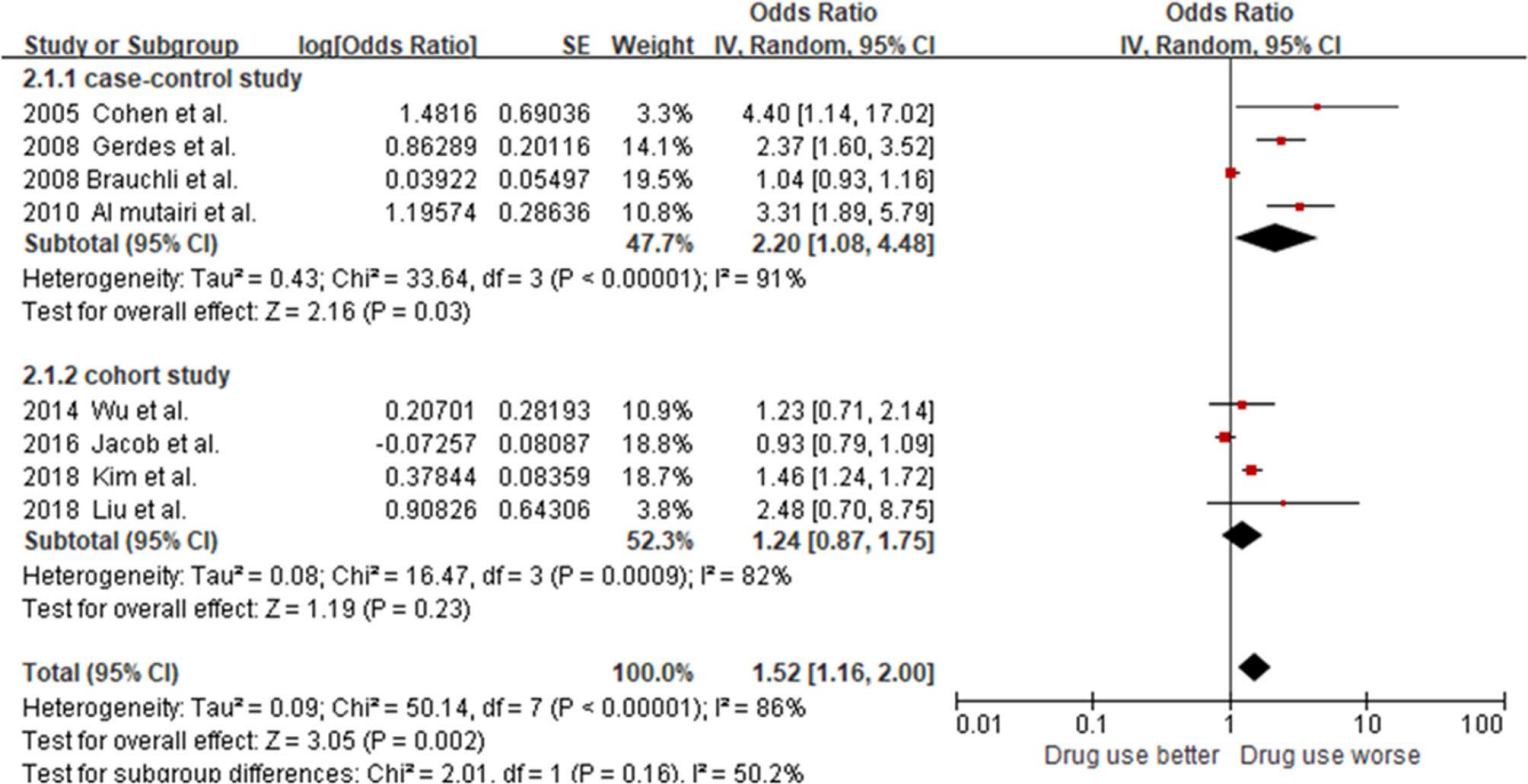
Forest plot to compare psoriasis risk among ACE inhibitor users vs non-users.

For the association of ACE inhibitor use with psoriasis incidence, eight studies with a total of 54,509 patients with a psoriasis diagnosis were included in the meta-analysis. As shown in Fig. 2, ACE inhibitor user group had a higher association with psoriasis incidence compared to the non-user group (Odds ratio (OR) 1.52, 95% confidence interval (CI) 1.16–2.00). We observed heterogeneity between these studies (*I*^2^ = 86%; *p* < 0.00001); hence, the random-effects model was used to calculate effect size. As there were four studies each in case–control and cohort study, we performed a subgroup analysis by study design. The ORs were 2.20 (95% CI 1.08–4.48) for case–control studies and 1.24 (95% CI 0.87–1.75) for cohort studies.

**Figure 3:**
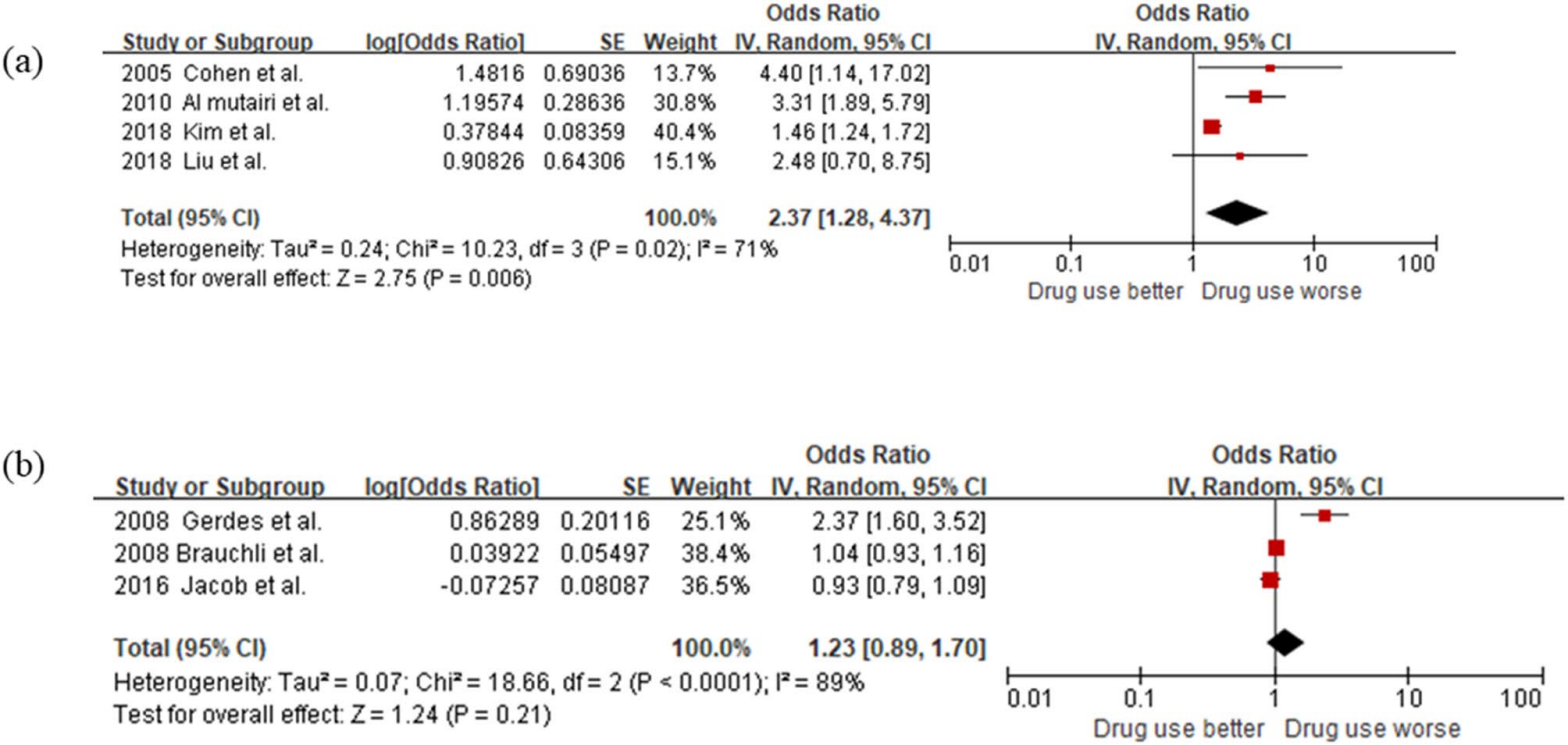
Forest plots to compare psoriasis risk among ACE inhibitor users versus non-users by continent. (**a**) Asia. (**b**) Europe.

For a subgroup analysis by continent, we divided into two groups; four and three studies in Asia^11,14,17,18^ and Europe^12,13,16^, respectively. As shown in Fig. 3, the analysis of the Asia subgroup showed that the ACE inhibitor user group had a higher risk of psoriasis incidence than the non-user group (OR 2.37, 95% CI 1.28–4.37), whereas the analysis of the Europe subgroup failed to show statistical significance. OR of the analysis of Asia was higher than that of all 8 studies (2.37 vs 1.52). Further analysis using subgroups of the West Asia and the East Asia showed that higher association between ACE inhibitor use and psoriasis incidence was found in studies conducted in West Asia (OR 3.45, 95% CI 2.05–5.79) compared to those in East Asia (OR 1.47, 95% CI 1.25–1.73).

**Figure 4:**
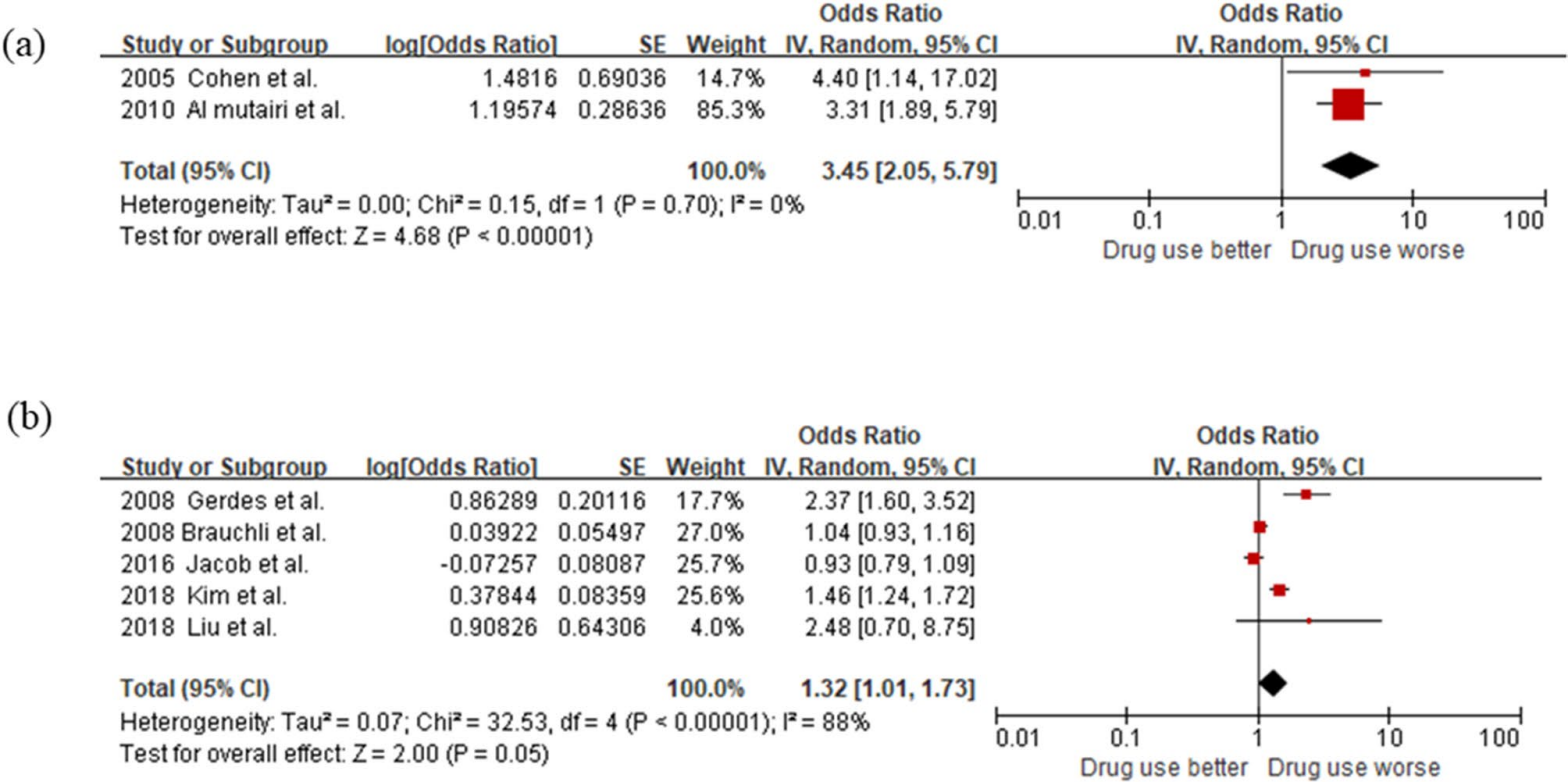
Forest plots to compare psoriasis risk among ACE inhibitor users verrsus non-users by climate. (**a**) Dry. (**b**) Temperate.

For the subgroup analysis by climate, since there were five and two were conducted in temperate climate^12,13,16–18^ and dry climates^11,14^, respectively. As shown in Fig. 4, the ACE inhibitor users within the dry climate subgroup showed a considerably higher risk of psoriasis incidence compared to the non-users (OR 3.45, 95% CI 2.05–5.79), whereas the analysis of the temperate climate subgroup showed marginal significance (*p* = 0.05).

### Sensitivity analysis and publication bias

Neither the Egger’s test (z = 0.68, *p* = 0.50) nor the Begg’s test (t = 1.06, *p* = 0.40) showed significant publication bias for these studies. Sensitivity analysis was performed by sequentially excluding each study. There were no significant effects on ORs for the association of ACE inhibitor use with psoriasis and heterogeneity (OR 1.36–1.74, *I*^2^ range 84%-88%).

## Discussion

The results of our meta-analysis indicate a significant association between ACE inhibitor use and psoriasis incidence. The sensitivity analyses yielded similar results, demonstrating the robustness of the results. In the subgroup analyses by continent and climate, risk of psoriasis related to ACE inhibitor use was higher in Asia and in a dry climate. Although statistical significance was obtained in the subgroup analysis using case–control studies, we failed to get a statistical significance in cohort studies. This was possibly due to the Jacob et al. study tending to be contrary to other studies.

A hypothesis for the association between ACE inhibitor use and psoriasis is related to bradykinin. ACE has two natural substrates and two catalytic domains: cleaving angiotensin I and inactivating bradykinin^19^. An in vitro study showed that ACE inhibitors had higher affinity to the bradykinin than the angiotensin I binding sites, indicating that these agents primarily inhibit bradykinin degradation^20^. The increased bradykinin levels because of ACE inhibitor use can cause vascular relaxation through the release of endothelial relaxation factors including prostacyclin, nitric oxide (NO), and epoxyeicosatrienoic acids. In contrast, inhibition of bradykinin degradation by ACE inhibitors alters the kinin-kallikrein arachidonic acid system leading to increased concentrations of inflammatory metabolites. This kinin-kallikrein arachidonic acid system is an important pathway in the pathogenesis of psoriasis^20–23^.

Angiotensin II is known to have a proinflammatory activity, which is mediated by the activation of macrophages, T-cells, mesangial cells, dendritic cells, and vascular smooth muscle cells^24^. Moreover, angiotensin II stimulates the production of specific cytokines such as interleukin-12, potent monocyte chemoattractant protein-1, NO, and tumor necrosis factor-α, thereby causing infiltration of inflammatory cells into tissues^24,25^. A previous in vivo study observed that angiotensin II enhances vascular inflammation by activating nuclear factor-kappa B (NF-κB)-mediated pro-inflammatory genes. Angiotensin II also downregulates peroxisome proliferator-activated receptors (PPARs) alpha and gamma, resulting in decrease of the anti-inflammatory potential of PPARs^26^.

The increased risk of psoriasis by the use of ACE inhibitors, although angiotensin II has aforementioned proinflammatory properties, is partially explained by the various activities of angiotensin II receptors. Angiotensin II has two major G protein-coupled receptor subtypes, the angiotensin II type 1 receptor (AT1R) and the angiotensin II type 2 receptor (AT2R)^27^. Binding of angiotensin II to AT1R increases inflammatory response whereas binding to AT2R decreases it. A recent study demonstrated that AT2R stimulation reduced expression of proinflammatory cytokine genes and activation of NF-κ in rheumatoid synovitis^28^. The use of ACE inhibitors prevents angiotensin II from binding to both receptors. Therefore, psoriasis due to ACE inhibitor use could involve more complex processes. A further study of this pathway is needed.

A previous study showed that Chinese patients had a notably higher risk of ACE inhibitor-related cough than Caucasians^29^. In a meta-analysis, the incidence of cough due to ACE inhibitor use has been reported to be 2.7-fold higher in East Asian populations compared to that in Caucasian populations^30^. These findings suggest the possible association between race and ACE inhibitor-related cough. Because bradykinin is seemingly the main mechanism of both ACE inhibitor-induced psoriasis and cough, the higher incidence of ACE inhibitor-related cough in Asians could explain the high risk of psoriasis in Asians.

Psoriasis is a multifactorial disease with both genetic and environmental contributing factors to its clinical manifestation^31^. In general, psoriasis is more common in colder northern regions than in tropical regions. Incidence among African blacks from the hot, wet west-central countries is less than that among African blacks from the milder, dry east-central nations^31^. Consistent with this finding, the climate subgroup analysis in our study showed a stronger association between ACE inhibitor use and psoriasis in dry climates.

This meta-analysis has some limitations that should be considered when interpreting the results. First, considerable heterogeneity was observed. Second, some confounding factors, which could affect the risk of psoriasis, could not be adjusted for. For example, most patients using ACE inhibitors for hypertension or other indications generally have more than one commitment medications, which could affect study results. Besides, within-study variation remained even after subgroup analysis due to the lack of adjusted odds ratio data in the included studies. Third, the duration and dosage of the drug were not specified. Fourth, the power of Begg's and Egger's test was low due to the number of included studies was small (less than 10). Therefore, the results of Begg's and Egger's test need to be interpreted with caution. Although we conducted subgroup analyses to look into the between-study variations, they still have within-study variations.

Nevertheless, this study is valuable in that it is the first systematic review and meta-analysis to evaluate the association between ACE inhibitor use and psoriasis. By combining the statistically insignificant findings of several studies, this study revealed significant associations between ACE inhibitor use and psoriasis. More prominent association between ACEI inhibitor use and psoriasis incidence was found in studies conducted in Asia or dry climate area. Based on our results, ACE inhibitor users should be carefully screened for cutaneous adverse events, particularly psoriasis.

## Methods

### Literature search strategy

The literature search included querying PubMed, Embase, and Web of Science for studies up to August 6, 2020, on the association between ACE inhibitor use and psoriasis incidence. The search included the following keywords: ((psoriasis OR psoria* OR psoriatic) AND ((ACEI OR ACE inhibitor* OR angiotensin converting enzyme inhibitor*) OR (antihypertensive agent* OR antihypertensive drug* OR antihypertensive medication*)). After removing duplicates, two researchers independently screened the titles and abstracts of all records to identify potentially eligible studies. Then, a full-text review was performed to determine final inclusion according to the eligibility criteria. In cases of disagreement, a consensus was reached by discussion.

### Inclusion and exclusion criteria

The following criteria were used to identify eligible studies: (1) selected participants with psoriasis diagnosed using specific criteria and controls without psoriasis; (2) used case–control or cohort study designs; (3) provided sufficient information to calculate OR or hazard ratio (HR) and a 95% CI; and (4) published in English. Exclusion criteria included (1) conference or meeting abstracts, summaries, reviews, comments, letters, news, case reports, case series, and editorials; (2) studies without appropriate data; (3) studies that did not confirm whether drugs were taken before diagnosis; (4) studies not medically classified as psoriasis; or (5) repeated study population.

### Data extraction

The following information was extracted from each study: name of the first author, publication year, country, study design, definition of patients with psoriasis, the number of patients with psoriasis (male %), the population size (male %), OR or HR with 95% CI, and comorbidities when available.

### Quality assessment

NOS was used to assess the quality of selected studies^32^. The scoring system of this scale was based on three components: subject selection (0–4 points); comparability of study groups (0–2 points); and exposure (case–control study) or outcome (cohort study; 0–3 points). The highest NOS score available for each publication was 9 points.

### Statistical analysis

We used ORs with 95% CIs as measures of effect size of ACE inhibitors for psoriasis. Statistical significance was analyzed using the z test, and a *p* value < 0.05 was considered statistically significant. It was assumed that the OR calculated from case–control studies approximates the HR in cohort studies. Heterogeneity was assessed using the Q test and *I*^2^ test^33^. Based on the heterogeneity results, either the fixed-effects model (Mantel–Haenszel method) or the random-effects model (when heterogeneity existed [*p* < 0.1, *I*^2^ > 50%]; DerSimonian-Laird method) was used to calculate effect size^34,35^.

The Egger regression test of the funnel plot was performed to identify publication bias^36^. A *p* value < 0.05 was considered statistically significant. Sensitivity analysis using the leave-one-out method was performed to assess the stability of results. Subgroup analyses by study design, continent and climate were also conducted if there were at least two studies in each subgroup. All statistical analyses were performed using Review Manager (RevMan) version 5.4 (The Cochrane Collaboration, Copenhagen, Denmark) and R software (version 3.6.0; R Foundation for Statistical Computing, Vienna, Austria). The paper was written according to the Preferred Reporting Items for Systematic Reviews and Meta-Analyses (PRISMA) guidelines^37^.
